# Hypoxia-induced metabolic shifting and AHR/BCRP axis impairment drive placental dysfunction in early-onset preeclampsia and FGR

**DOI:** 10.3389/ftox.2026.1782187

**Published:** 2026-06-22

**Authors:** Jingjing Shi, Wenxuan Zhu, Haixia Liu, Ping Yang, Yongzhong Gu, Wen Peng, Jinlai Meng

**Affiliations:** 1 Department of Obstetrics and Gynecology, Shandong Provincial Hospital Affiliated to Shandong First Medical University, Jinan, Shandong, China; 2 Jining Medical University, Jining, Shandong, China; 3 Key Laboratory of Maternal & Fetal Medicine of National Health Commission of China, Shandong Provincial Maternal and Child Healthcare Hospital Affiliated to Qingdao University, Jinan, Shandong, China; 4 Department of Obstetrics and Gynecology, Shandong Provincial Hospital, Shandong University, Jinan, Shandong, China

**Keywords:** aryl hydrocarbon receptor (AHR), breast cancer resistance protein (BCRP), fetal growth restriction (FGR), hypoxia-inducible factor 1 alpha (HIF-1α), placental barrier, preeclampsia

## Abstract

**Background:**

Placental hypoxia and vascular dysfunction are hallmarks of preeclampsia (PE). The Breast Cancer Resistance Protein (BCRP) is critical for maintaining the placental barrier against oxidative stress. We investigated whether the hypoxic microenvironment drives BCRP dysregulation and how this correlates with fetal growth restriction (FGR) in severe preeclampsia.

**Methods:**

This case-control study enrolled 59 normotensive and 72 preeclamptic pregnancies to compare clinical phenotypes and placental BCRP expression across distinct gestational age subgroups. Mechanistic investigations were conducted using human BeWo trophoblasts under pathological hypoxia (1%O_2_​). We integrated metabolic profiling (glycolytic shift and JC-1 mitochondrial assays) with rigorous molecular techniques—including Co-immunoprecipitation (Co-IP) and subcellular fractionation—to elucidate the competitive crosstalk between HIF-1α and AHR, further validating barrier impairment via Hoechst 33342 functional efflux assays.

**Results:**

Clinically, patients with early-onset preeclampsia (28–33^+6^ weeks) exhibited a markedly higher incidence of FGR compared to late-onset or control groups. In these compromised placentas, BCRP expression was significantly suppressed and inversely correlated with the hypoxia marker GLUT1. *In vitro* assays delineated a “dual-hit” pathological cascade triggered by chronic hypoxia. First, trophoblasts underwent severe metabolic reprogramming characterized by massive glycolytic upregulation and mitochondrial dysfunction. Second, stabilized HIF-1α competitively sequestered the obligate co-receptor ARNT, systematically dismantling the AHR/ARNT transcriptional complex. This specific molecular “competitive binding” impaired AHR-mediated protective signaling, resulting in a critical loss of functional BCRP efflux capacity.

**Conclusion:**

Our study defines a distinct “dual-hit” molecular phenotype in early-onset preeclampsia. Chronic hypoxia not only drives an acute metabolic crisis but also actively dismantles the protective AHR/BCRP placental barrier via HIF-1α-mediated ARNT sequestering. This synergistic failure compromises placental xenobiotic detoxification, exacerbates fetal toxicological vulnerability, and fundamentally contributes to the profound severity of FGR.

## 1 Introduction

Preeclampsia (PE) is a severe, pregnancy-specific syndrome driven by placental vascular maladaptation, representing a leading cause of maternal and perinatal morbidity (Rana et al., 2019; Burton et al., 2019). While the clinical manifestations are systemic, early-onset PE is fundamentally rooted in defective trophoblast invasion and failed spiral artery remodeling. This structural failure prevents the physiological transition from a hypoxic to a normoxic maternal-fetal interface, trapping the developing placenta in a state of chronic, pathological ischemia beyond the first trimester (Laresgoiti-Servitje and Gomez-Lopez, 2012; Kojovic et al., 2020; Poon et al., 2019; Genbacev et al., 1997; Zhao et al., 2021; Caniggia et al., 2000). Despite its prevalence, the molecular etiology translating this prolonged hypoxic stress into devastating clinical outcomes—most notably, the exceptionally high incidence of Fetal Growth Restriction (FGR) predominantly observed in early-onset disease (<34 weeks of gestation)—remains incompletely delineated (ACOG Practice Bulletin No.202, 2019).

The primary cellular response to this hostile microenvironment is orchestrated by the oxygen sensor Hypoxia-inducible factor 1-alpha (HIF-1α). Aberrant HIF-1α stabilization triggers the dramatic upregulation of Glucose Transporter 1 (GLUT1) alongside a robust cascade of rate-limiting glycolytic enzymes (e.g., HK2, PFK1, LDHA). While this acute shift toward anaerobic glycolysis initially limits cell death, this massive metabolic reprogramming inevitably uncouples oxidative phosphorylation and drives mitochondrial dysfunction (Ietta et al., 2006; Francois et al., 2017; Odibo et al., 2011; Wei and Hu, 2014). This profound intracellular crisis comes at a significant functional cost, establishing the primary “first hit” in the pathological cascade of early-onset disease.

We specifically sought to investigate how this hypoxic metabolic shift fundamentally dismantles placental barrier functionality—representing a substantial “second hit.” The Breast Cancer Resistance Protein (BCRP/ABCG2) is a paramount efflux transporter at the maternal-fetal interface, essential for actively shielding the fetus from targeted maternal xenobiotics, environmental toxins, and endogenous oxidative stress (Mao, 2008; Than et al., 2015; Mao and Unadkat, 2015; Staud et al., 2006; Cygalova et al., 2009). Intriguingly, in stark contrast to the elevation of hypoxic markers, placental BCRP expression heavily collapses under hypoxic conditions akin to early-onset PE (Francois et al., 2017; Lye et al., 2013). Because the master transcriptional regulator of BCRP, the Aryl Hydrocarbon Receptor (AHR), strictly relies on the obligate cofactor ARNT (HIF-1β) to initiate downstream transcription (Tan et al., 2010; Vorrink and Domann, 2014)—the exact same heterodimeric partner required by HIF-1α—this parallel downregulation strongly suggests an underlying regulatory conflict.

We hypothesize an intracellular competitive sequestration mechanism, whereby the massive, hypoxia-induced accumulation of HIF-1α rapidly depletes the available nuclear ARNT pool. We propose that this core “molecular Sequester” functionally silences the AHR pathway and profoundly suppresses BCRP (Tompkins et al., 2010; Ebert et al., 2005). To test this hypothesis, we systematically evaluated a well-characterized clinical cohort rigidly stratified by gestational age (spanning early-onset 28–33^+6^ weeks to term) to correlate critical FGR thresholds with clinical severity. We subsequently coupled these clinical observations with rigorous *in vitro* validations. By employing extensive structural and metabolic profiling, alongside targeted subcellular (nuclear/cytosolic) fractionation, we sought to capture physical evidence of this molecular Sequester. Ultimately, we aim to thoroughly define this distinct “dual-hit” molecular phenotype, demonstrating how HIF-1α-driven “disruption of the AHR/ARNT complex” and marked reduction exacerbate the toxicological vulnerability of the developing fetus.

## 2 Methods

### 2.1 Study design and clinical participants

This study was conducted at the Department of Obstetrics, Shandong Provincial Hospital. The study population comprised 131 participants, including 59 women with normal uncomplicated term pregnancies (Control group; mean age: 33.5 ± 3.5 years; mean gestational age: 38.3 ± 0.8 weeks) and 72 patients diagnosed with preeclampsia (PE group; mean age: 35.2 ± 4.7 years; mean gestational age: 32.8 ± 3.4 weeks).

Preeclampsia was diagnosed according to the guidelines of the American College of Obstetricians and Gynecologists (ACOG). The criteria included systolic blood pressure ≥140 mmHg and/or diastolic blood pressure ≥90 mmHg on two occasions at least 4 h apart after 20 weeks of gestation, accompanied by proteinuria (≥0.3 g/24 h or ≥2+ on dipstick analysis). In the absence of proteinuria, diagnosis was confirmed by new-onset hypertension with severe features, including thrombocytopenia, renal insufficiency, impaired liver function, pulmonary edema, or new-onset cerebral/visual disturbances. Exclusion criteria encompassed: (1) multiple gestations; (2) conception via assisted reproductive technology; (3) chronic/secondary hypertension; (4) pre-existing conditions (renal, diabetic, cardiac, or autoimmune diseases); (5) acute/chronic infections; and (6) known fetal anomalies.

Fetal Growth Restriction (FGR) was defined as a birth weight below the 10th percentile for gestational age and sex, according to the American College of Obstetricians and Gynecologists (ACOG) criteria (ACOG, Practice Bulletin No202, 2019).

This study was approved by the Ethics Committee of Shandong First Medical University, and written informed consent was obtained from all participants prior to enrollment.

### 2.2 Clinical data and sample collection

Clinical parameters, including age, BMI, gestational age, mode of delivery, and neonatal outcomes (birth weight and Apgar scores), were recorded. Peripheral venous blood samples (5 mL) were analyzed at the Department of Clinical Laboratory to quantify platelet count, neutrophil-to-lymphocyte ratio (NLR), serum creatinine, BUN, ALT, and AST. Placental tissue samples were immediately dissected after delivery, rinsed in sterile saline, and processed for subsequent immunohistochemical or molecular biological analyses.

### 2.3 Immunohistochemistry (IHC)

IHC staining was evaluated semi-quantitatively using the Fromowitz scoring system (Fromowitz et al., 1987), which integrates both staining intensity and the percentage of positive cells. Five high-power fields were randomly selected for each section. Staining intensity was graded as: 0 (no staining), 1 (weak/light yellow), 2 (moderate/yellow-brown), and 3 (strong/brown). The proportion of positive cells was scored as: 1 (≤25%), 2 (26%–50%), 3 (51%–75%), and 4 (>75%). The final Fromowitz score (range 1–12) was calculated by adding the intensity score to the proportion score. Cells exhibiting distinct brown staining in the cytoplasm or cell membrane were considered positive.

### 2.4 Cell culture and hypoxic *in vitro* modeling

The human placental choriocarcinoma cell line BeWo (Cat#: iCell-h403) was authenticated and obtained from iCell Bioscience Inc (Shanghai, China). Cells were maintained in RPMI-1640 medium supplemented with 10% fetal bovine serum (FBS) and cultured at 37 °C in a standard 5% CO_2_​ incubator. Upon reaching 80%–90% confluence, cells were passaged using 0.25% Trypsin-EDTA.

For *in vitro* hypoxic modeling, BeWo cells were seeded into culture dishes and synchronized using reduced serum medium (1% FBS) prior to treatments. Cells were randomly assigned to normoxia (Control, 20% O2​) or severe hypoxia groups (Hyp 24 h and Hyp 48 h). Hypoxic exposure was strictly controlled using a dedicated tri-gas incubator (Thermo Fisher Scientific) maintained at 1% O_2_, 5% CO_2_, and 94% N_2_ at 37 °C.

### 2.5 Western blot analysis

Total cellular protein was extracted using RIPA lysis buffer (P0013C, Beyotime, China) supplemented with a protease and phosphatase inhibitor cocktail (P1006, Beyotime). Protein concentrations were quantified via the BCA assay (P0012, Beyotime). Equal amounts of denatured protein (boiled at 99 °C for 10 min, excluding GLUT1 samples to prevent aggregation) were separated by 8%–10% SDS-PAGE and transferred onto PVDF membranes (IPVH00010, Millipore).

After blocking for 2h, membranes were incubated overnight at 4 °C with specific primary antibodies. The primary antibodies included specific clones targeted for rigorous validation: ABCG2/BCRP (Clone D5V2K, #42078T, CST), GLUT1 (Clone E4S6I, #73015T, CST), HIF-1α (Clone PT0978R, YM8735, Immunoway), HIF-1β/ARNT (Clone PT1272R, YM9114, Immunoway), AHR (Clone PT1498R, YM9340, Immunoway), Hexokinase II (Clone PT0189R, YM8118, Immunoway), PFKM (Clone PT 1820R, YM960063, Immunoway), Lactate Dehydrogenase (Clone PT0186R, YM811, Immunoway), PDK1 (Clone PT1266R, YM9108, Immunoway), and Histone H3 (Clone PT0508R, YM8335, Immunoway). Following thorough washing in TBST, membranes were incubated with respective HRP-conjugated secondary antibodies (1:5000, ZSGB-BIO) for 1.5 h at room temperature. Chemiluminescence was developed using ECL substrate and visualized with the Tanon 4600 Imaging System.

### 2.6 Subcellular (nuclear/cytosolic) fractionation

To characterize the intracellular localization and nuclear translocation of target transcription factors (HIF-1α and AHR), subcellular fractionation was performed utilizing a Nuclear and Cytoplasmic Protein Extraction Kit (P0028, Beyotime) according to the manufacturer’s protocol. Briefly, harvested cells were lysed in hypotonic buffer containing protease inhibitors, and cytoplasmic fractions were collected after centrifugation. Intact nuclei were subsequently lysed using a high-salt nuclear extraction buffer. Equivalent amounts of nuclear and cytosolic proteins were analyzed via Western blot, utilizing Histone H3 and GAPDH/β-actin as exclusive compartmental loading controls, respectively.

### 2.7 Co-immunoprecipitation (Co-IP) assay

To validate the competitive physical interactions between intracellular transcription factors, Co-IP was carried out using a Protein A Agarose Immuno-precipitation Kit (P2193M, Beyotime). Cell lysates containing equal amounts of protein were pre-cleared and subsequently incubated overnight at 4 °C with the indicated trapping antibody on a gentle rotator. Protein A agarose beads were then added to capture the immune complexes. After extensive washing to remove nonspecific binding, the precipitated protein complexes were eluted by boiling in 5× SDS loading buffer and subjected to standard Western blot analysis to identify the co-precipitated associated partners (e.g., AhR, HIF-1α, and HIF-1β/ARNT).

### 2.8 Extracellular metabolic profiling (glucose and lactate assays)

To quantify the hypoxia-induced glycolytic shift, the culture supernatant of BeWo cells was collected post-treatment. Glucose consumption was determined using a GOD-POD colorimetric assay kit (E-BC-K234-M, Elabscience), and L-Lactate production was measured using a specific colorimetric assay kit (E-BC-K044-M, Elabscience). Absorbance values were evaluated utilizing a full-wavelength microplate reader (Thermo Fisher Scientific) following the manufacturer’s instructions, and metabolic concentrations were normalized to corresponding cellular protein contents.

### 2.9 Mitochondrial membrane potential (ΔΨm) measurement

Mitochondrial dysfunction was assessed using the JC-1 fluorescent probe (C2006, Beyotime). Harvested BeWo cells were re-suspended and stained with JC-1 working solution for 20 min at 37 °C in the dark. The cells were then washed and analyzed flow cytometrically (BD Biosciences). The transition from JC-1 aggregates (detectable in the PE channel/red fluorescence) to monomers (detectable in the FITC channel/green fluorescence) was utilized as an indicator of mitochondrial membrane potential impairment.

### 2.10 Hoechst 33342 functional efflux assay

The physiological functionality of the BCRP transporter in the placental barrier model was evaluated through a direct fluorescent substrate efflux assay using Hoechst 33342 (C1022, Beyotime). Harvested cells were pre-incubated with PBS containing 5 μM Hoechst 33342 at 37 °C for 30 min to ensure substrate loading. A portion of the cells was immediately washed and transferred to a microplate to measure initial intracellular accumulation (RFU_UPTAKE_) at Ex/Em 350 nm/461 nm. The remaining cells were incubated in dye-free PBS for an additional 1 h at 37 °C, permitting BCRP-mediated substrate transport. Following incubation, retention fluorescence was documented (RFU_EFFLUX_), and the relative efflux capacity was calculated using the equation:

$$\text{Efflux Rate}\left(\%\right)=\left({\text{RFU}}_{\text{UPTAKE}}-{\text{RFU}}_{\text{EFFLUX}}\right)/{\text{RFU}}_{\text{UPTAKE}}\times 100\%$$

### 2.11 Statistical analysis

Statistical analyses were executed using SPSS version 25.0 (IBM Corp., NY) and GraphPad Prism 8.0 (GraphPad Software, CA). Quantitative data are presented as the mean ± standard deviation (SD) of at least three independent biological replicates. The normality of data distribution was assessed using the Shapiro-Wilk test. For normally distributed data, differences between two groups were assessed using Student’s t-test, whereas comparisons among multiple groups were determined via one-way analysis of variance (ANOVA) followed by Tukey’s *post hoc* test for multiple comparisons. For non-normally distributed data or unequal variances, appropriate non-parametric tests (such as the Mann-Whitney U test) or Welch’s ANOVA were applied. Correlation between clinical variables was analyzed using Spearman’s rank correlation coefficient. A P-value <0.05 was considered statistically significant. To account for potential confounding factors, specifically the significant difference in gestational age between the control and PE groups, a multivariate linear regression analysis was performed with BCRP expression as the dependent variable and gestational age and group status as independent variables.

## 3 Results

### 3.1 Demographic and clinical profile

The study comprised 131 participants (59 Control, 72 PE). Groups were matched for age and parity, though pre-pregnancy BMI was significantly higher in the PE group (P = 0.003; Table 1). The PE group presented with severe clinical features, including hypertension and significant elevations in liver (ALT, AST) and kidney (Cr, BUN) function tests (P < 0.001; Table 2). Obstetric outcomes were notably compromised in the PE group, characterized by earlier gestational age at delivery and reduced birth weight (P < 0.001; Table 3).

**TABLE 1:** General information.

Characteristics	Normal term pregnancy	Pre-eclampsia	P value
Maternal age (year)	33.5 ± 3.5	35.2 ± 4.7	0.069
BMI(kg/m^2^)	29.5 ± 3.55	32.61 ± 5.23	0.003*
Primiparous women (%)	19.6	13.9	0.484

BMI, body mass index; Primiparous women (%): The proportion of pregnant women with their first pregnancy.

*P<0.05 means statistically significant.

**TABLE 2:** Clinical data.

Characteristics	Normal term pregnancy	Pre-eclampsia	P value
Systolic pressure (mmHg)	110.6 ± 11.0	153.9 ± 8.0	<0.001*
Diastolic pressure (mmHg)	73.3 ± 8.2	99.4 ± 7.6	<0.001*
Cr (μmol/L)	48.20 ± 7.68	69.39 ± 28.16	<0.001*
BUN(mmol/L)	3.18 ± 0.84	5.94 ± 2.0	<0.001*
ALT (U/L)	9.5 (7.0,12.0)	15.8 (11.9,25.4)	<0.001*
AST (U/L)	17.0 (15.0,20.3)	24.8 (19.5,29.6)	<0.001
PLT (10^9/L)	209.9 ± 51.0	187.3 ± 55.9	0.06

Cr, creatinine clearance; BUN, blood urea nitrogen; ALT, alanine transaminase; AST, aspartate transaminase; PLT, platelet count.

*p* Value are considered the correlation between normal term pregnancy group and pre-eclampsia group among various indicators; *p* < 0.05*means statistically significant.

**TABLE 3:** Pregnancy outcome.

Characteristics	Normal term pregnancy	Pre-eclampsia	P value
Termination pregnancy age (weeks)	38.3 ± 0.8	32.8 ± 3.4	<0.001*
Birth weight (g)	3332.6 ± 375.1	1985.9 ± 716.7	<0.001*
APGAR (points)	10 (10,10)	7.1 (8.8,9.5)	<0.001*

Apgar score is the degree of clinical evaluation of newborn infants asphyxia.

*p* Value are considered the correlation between normal term pregnancy group and pre-eclampsia group among various indicators; *p* < 0.05*means statistically significant.

### 3.2 Increased prevalence of FGR in early-onset preeclampsia

**FIGURE 1:**
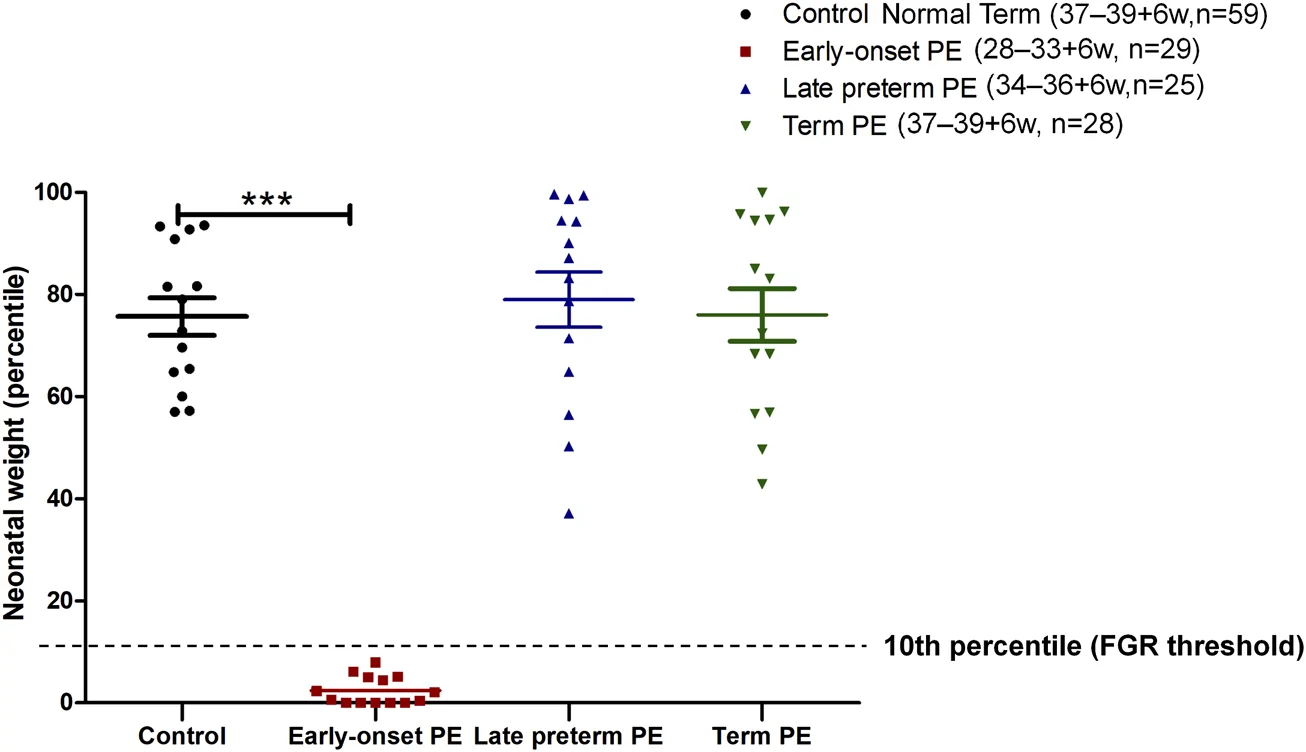
Prevalence of fetal growth restriction (FGR) stratified by gestational age and preeclampsia onset. FGR cases were identified based on birth weight below the 10th percentile for the respective gestational age. A significant elevation in FGR incidence was observed in the early-onset PE cohort (Group 1: 28–33^+6^ weeks, n = 29) relative to the normal term group (37–39^+6^ weeks, n = 59; **P < 0.001). Study groups: Control (n = 59); Group 1 (early-onset PE, n = 29); Group 2 (late-onset PE, 34–36^+6^ weeks, n = 25); Group 3 (term PE, 37–39^+6^ weeks, n = 28).

To evaluate the impact of preeclampsia on fetal development, we assessed the incidence of fetal growth restriction (FGR) defined as a birth weight below the 10th percentile according to ACOG criteria (ACOG practice bulletin no. 202, 2019). Clinical stratification demonstrated that FGR was disproportionately prevalent in the early-onset subgroup (28–33^+6^ weeks, n = 29), with an incidence significantly higher than those observed in the late-preterm (34–36^+6^ weeks) or term PE subgroups (Figure 1, P < 0.001). These findings suggest that the 28–33^+6^ week gestational window represents a period of heightened vulnerability to placental-mediated growth impairment in preeclamptic pregnancies.

### 3.3 Placental BCRP downregulation correlates with hypoxia-induced metabolic stress

**FIGURE 2:**
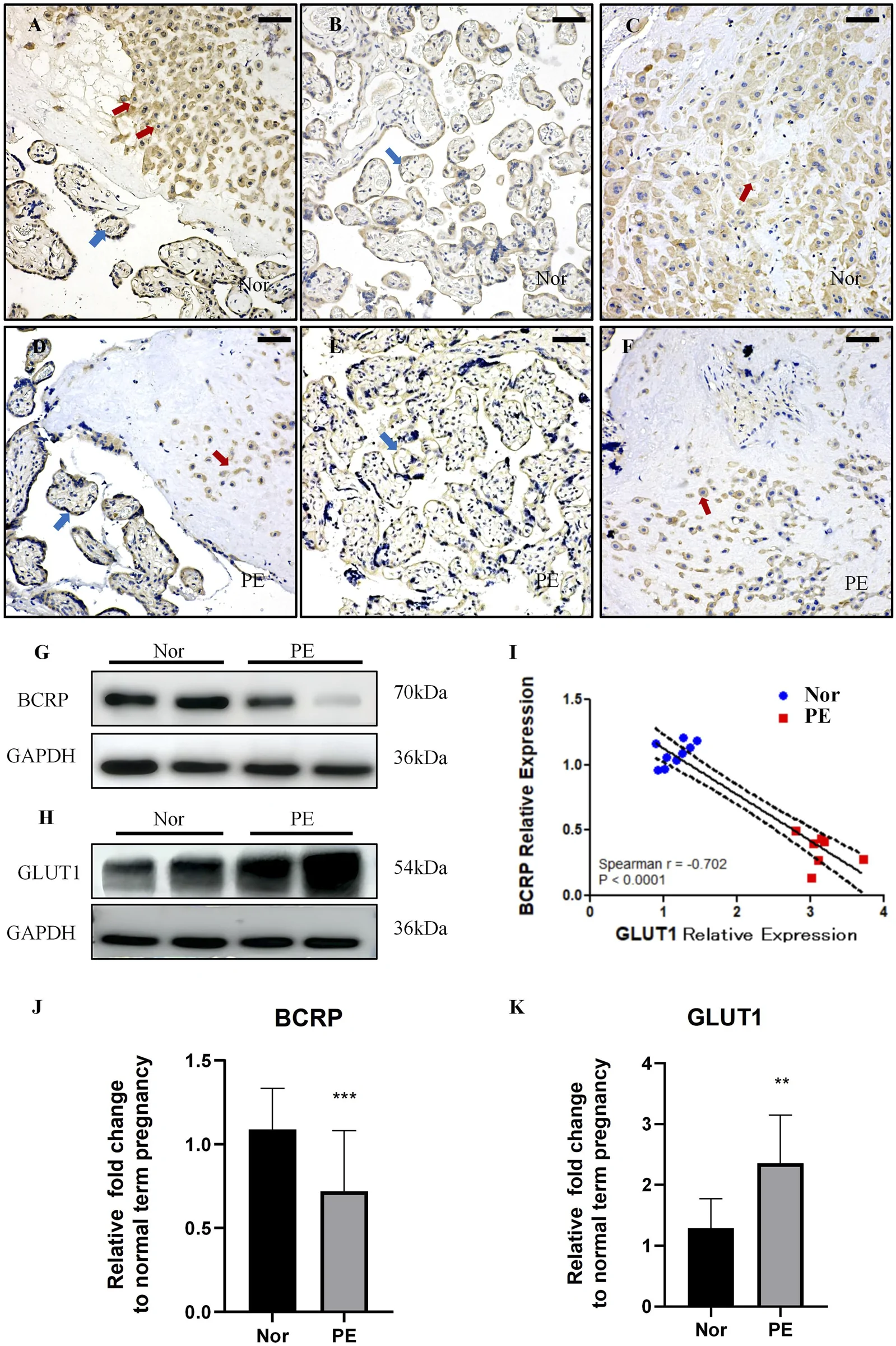
Reciprocal expression of BCRP and GLUT1 in normal and preeclamptic (PE) placentas. **(A–F)** Representative immunohistochemical (IHC) staining of BCRP in term control **(A–C)** and early-onset PE **(D–F)** placental villi (n = 20 per group). Red arrows indicate specific BCRP immunoreactivity in the syncytiotrophoblast layer; blue arrows denote the villous stroma. Scale bars = 20 μm. **(G,H)** Representative immunoblots for BCRP **(G)** and GLUT1 **(H)** in normal (Nor) versus PE placental tissues. GAPDH was used as the loading control **(I)** Correlation analysis showing a strong inverse relationship between placental BCRP and GLUT1 protein expression levels (n = 9; Spearman’s r = −0.702,P < 0.0001) **(J,K)** Densitometric quantification of BCRP **(J)** and GLUT1 **(K)** protein levels normalized to GAPDH. Data are presented as mean ± SD with individual data points superimposed. Intergroup differences were analyzed by Student’s t-test. **P < 0.01, ***P < 0.001 vs. Nor group.

We first examined the spatial distribution of BCRP in placental villi. Immunohistochemical (IHC) analysis revealed that BCRP was predominantly localized to the apical membrane of the syncytiotrophoblast, with significantly reduced staining intensity in the early-onset PE group compared to term controls (Figures 2A–F). This downregulation was confirmed by quantitative immunoblotting, which showed a significant decrease in BCRP protein levels in PE placentas (Figures 2G,J; P < 0.01).

To exclude the potential confounding effect of gestational age (GA), which differed significantly between the two groups (Table 3), we performed a multivariate linear regression analysis. PE status remained a significant independent predictor of BCRP expression (β = −0.698, P = 0.042; Table 4), whereas GA did not show a statistically significant independent effect P=0.473 . These results indicate that BCRP depletion is a specific pathological feature of pre-eclampsia rather than a secondary consequence of placental immaturity.

**TABLE 4:** Multivariate linear regression analysis of factors associated with placental BCRP expression.

Variable	Unstandardized β	Standard error (SE)	Standardized β	t	P-value
(Constant)	0.270	1.110	-	0.243	0.811
Gestational age	0.022	0.029	0.229	0.740	0.473
PE status	−0.495	0.219	−0.698	−2.254	*0.042**

*Statistically significant (*p* < 0.05). PE, status was coded as 0 = Control, 1 = PE.

Furthermore, the expression of GLUT1—a sensitive marker of chronic placental hypoxia—was significantly elevated in the same PE cohorts (Figures 2H,K; P < 0.001). Correlation analysis revealed a strong inverse relationship between BCRP and GLUT1 levels (Spearman’s r = −0.702, P < 0.0001; Figure 2I), suggesting that the loss of BCRP-mediated transport capacity is closely associated with the severity of placental hypoxic stress.

### 3.4 Hypoxia drives the reciprocal regulation of HIF-1α/glycolysis and AHR/BCRP pathways in vitro

**FIGURE 3:**
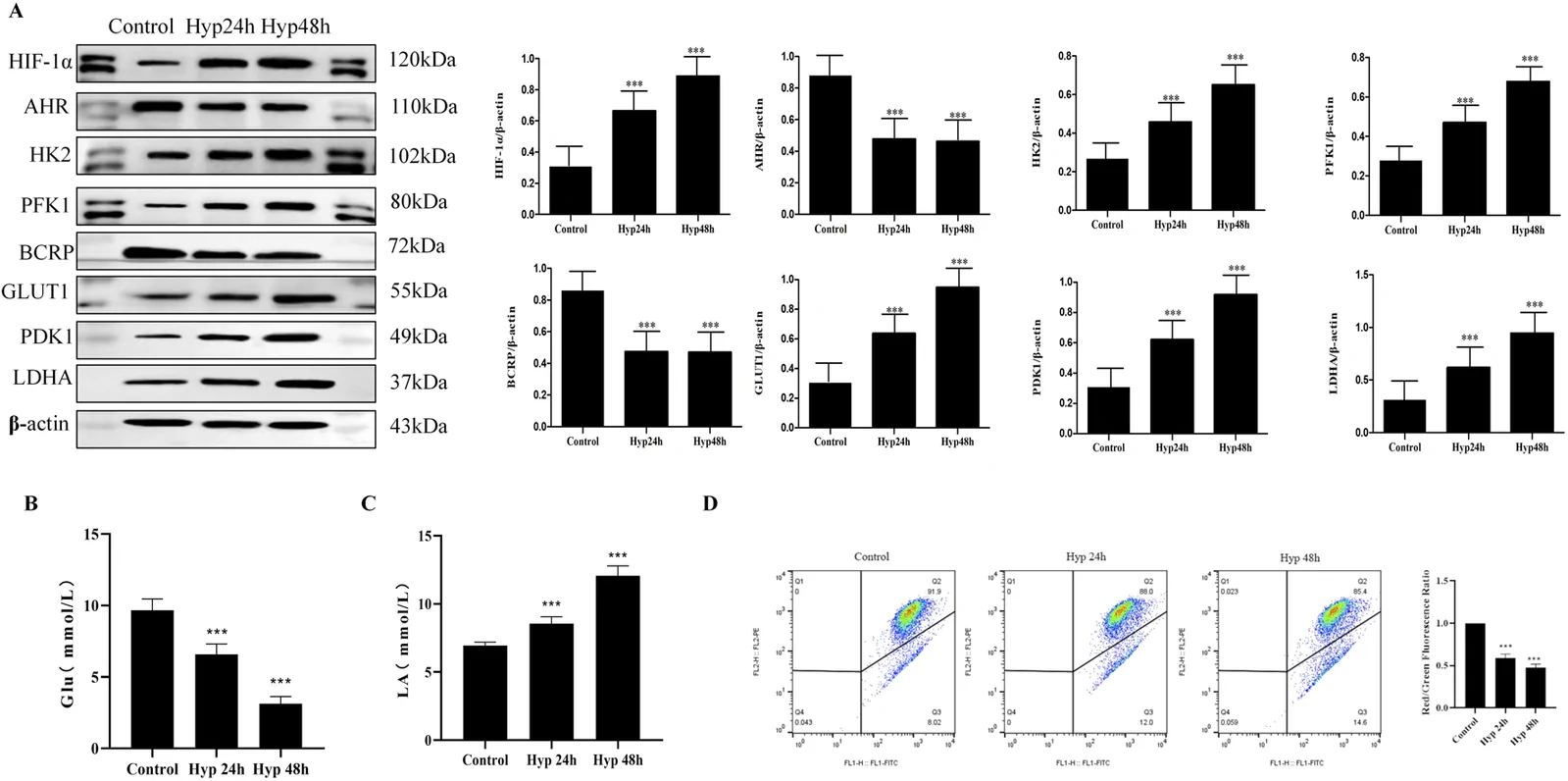
Hypoxia-induced metabolic reprogramming and mitochondrial dysfunction in trophoblasts. **(A)** Representative immunoblots (left) and corresponding densitometric quantification (right) showing the time-dependent effects of hypoxia (1%O2; 24h and 48 h) on core signaling regulators (HIF-1α, AHR), glycolytic enzymes (HK2, PFK1, PDK1, LDHA), the glucose transporter GLUT1, and the efflux transporter BCRP in BeWo cells. β-actin was used as the loading control. Hypoxia triggered a coordinated upregulation of the glycolytic machinery, concomitant with a significant suppression of the AHR-BCRP axis. Functional validation of the glycolytic shift via measurement of **(B)** glucose levels and **(C)** lactate production in the culture supernatant, demonstrating increased glucose consumption and glycolytic flux under prolonged hypoxic stress. **(D)** Assessment of mitochondrial membrane potential (Δψm) via JC-1 staining and flow cytometry. Representative dot plots (left) and quantified red-to-green fluorescence ratios (right) indicate progressive mitochondrial depolarization, confirming hypoxia-induced mitochondrial impairment. Data are presented as mean ± SD from at least three independent experiments. *P < 0.05, **P < 0.01, ***P < 0.001 vs. normoxic control.

To mechanistically link our clinical findings to hypoxic stress, we utilized a BeWo cell hypoxia model. Prolonged exposure to hypoxia (1% O_2_, 48 h) triggered a significant divergence in key signaling cascades: a robust activation of the HIF-1α axis, characterized by the upregulation of GLUT1 and essential glycolytic enzymes (HK2, PFK1, and LDHA), was observed alongside a profound suppression of the AHR/BCRP axis (Figures 3A,B). Functional assays further confirmed this metabolic shift, demonstrating enhanced glucose consumption and lactate secretion consistent with accelerated glycolytic flux (Figure 3C). Moreover, hypoxia-induced AHR downregulation directly correlated with the attrition of BCRP expression, mirroring the molecular phenotype observed in early-onset PE placentas. These *in vitro* results indicate that chronic hypoxia is a primary driver of the synchronized metabolic reprogramming and BCRP deficiency characteristic of placental insufficiency.

### 3.5 HIF-1α-mediated ARNT sequestration disrupts AHR nuclear translocation and placental barrier function

**FIGURE 4:**
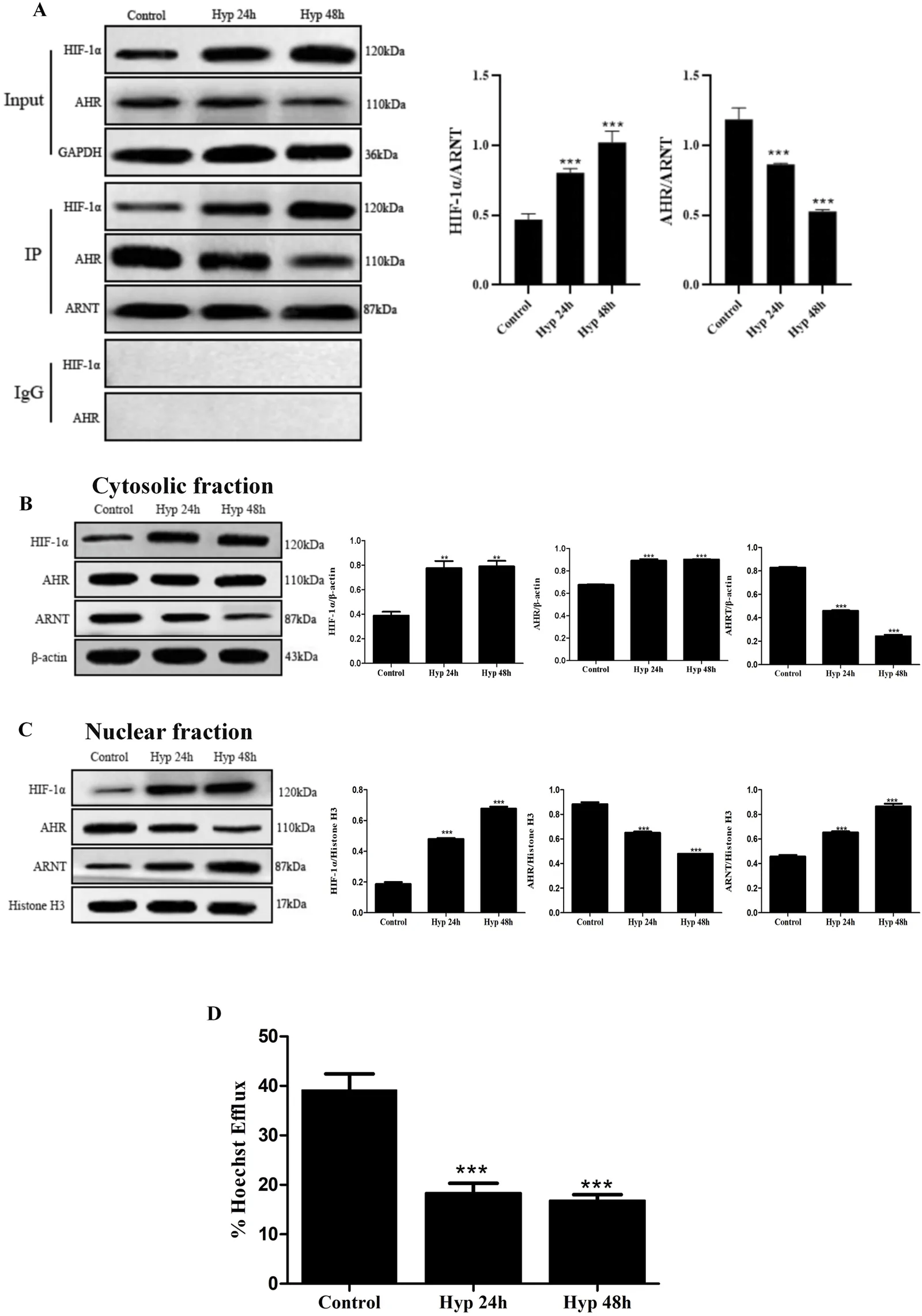
Hypoxia disrupts AHR nuclear localization and BCRP transporter function via HIF-1α-mediated ARNT sequestration. **(A)** Representative Western blots of Co-immunoprecipitation (Co-IP) assays characterizing the interactions among ARNT, HIF-1α, and AHR in BeWo cells cultured under normoxic or hypoxic conditions. Immunoprecipitation was performed using an anti-ARNT antibody, with non-specific IgG serving as a negative control. Accompanying bar graphs quantify the ratios of HIF-1α to ARNT and AHR to ARNT within the immunocomplexes. **(B,C)** Subcellular fractionation analysis evaluating the protein abundance of HIF-1α, AHR, and ARNT in the cytosolic **(B)** and nuclear **(C)** compartments. β-actin and Histone H3 served as loading controls for the cytoplasmic and nuclear fractions, respectively. Hypoxia triggered the simultaneous nuclear accumulation of HIF-1α and ARNT, concomitant with the restriction of AHR to the cytoplasm. **(D)** Functional assessment of BCRP efflux capacity using the specific fluorescent substrate Hoechst 33,342. The significant decline in the percentage of Hoechst efflux indicates the functional impairment of BCRP-mediated transport under sustained hypoxia. Data are presented as mean ± SD from at least three independent experiments. ***P < 0.001 vs. the normoxic control group.

To delineate the precise mechanism underlying BCRP downregulation, we investigated the competitive interplay between HIF-1α and AHR for their shared obligate heterodimeric partner, ARNT. Co-immunoprecipitation (Co-IP) assays revealed that under normoxia, ARNT preferentially bound to AHR. Conversely, hypoxic exposure significantly shifted this equilibrium, driving a robust interaction between HIF-1α and ARNT while simultaneously displacing AHR from the complex (Figure 4A).

This competitive sequestration profoundly impacted the subcellular distribution of these transcription factors. Subcellular fractionation demonstrated that hypoxia induced a massive nuclear accumulation of both HIF-1α and ARNT. Denied its binding partner, AHR was largely excluded from the nucleus and sequestered in the cytoplasm (Figures 4B,C), thereby disabling the AHR-driven transcriptional machinery essential for BCRP expression.

To determine if this molecular disruption translates to a loss of barrier function, we evaluated BCRP efflux activity using the fluorescent substrate Hoechst 33342. Consistent with the profound protein downregulation, sustained hypoxia significantly diminished the intracellular Hoechst efflux percentage (Figure 4D). Collectively, these findings establish that HIF-1α competitively intercepts ARNT, disrupting AHR/BCRP signaling and consequently compromising the functional integrity of the placental barrier.

### 3.6 Proposed “dual-hit” working model of trophoblast dysfunction in early-onset preeclampsia

**FIGURE 5:**
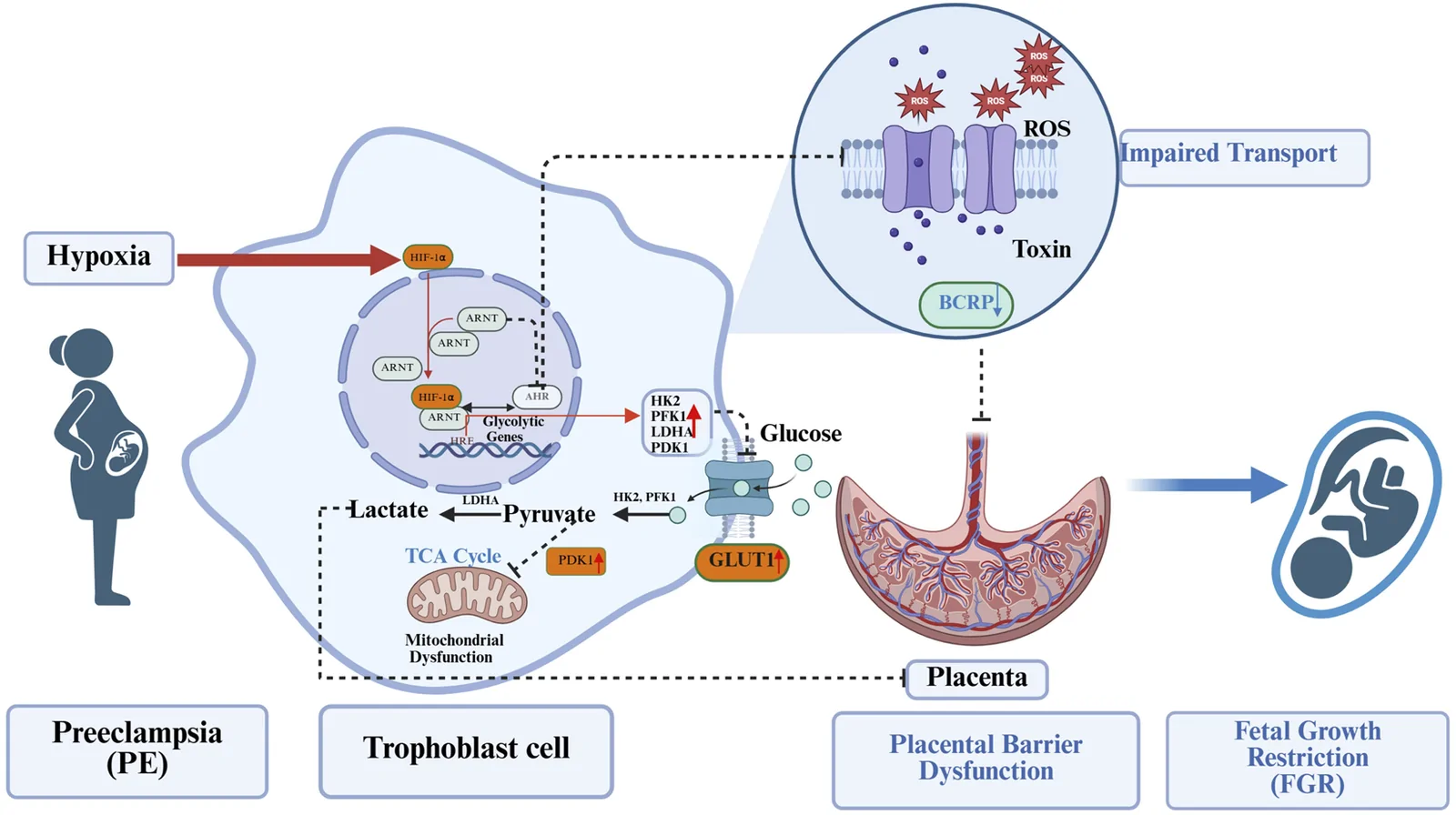
Schematic of the proposed “Dual-Hit” mechanism driving trophoblast dysfunction in early-onset preeclampsia. Under chronic placental hypoxia, stabilized HIF-1α accumulates in the nucleus and competitively sequesters ARNT (Hit 1: Barrier Impairment) This competitive sequestration deprives AHR of its obligate cofactor, thereby silencing AHR-mediated signaling and profoundly suppressing the transcription of the efflux transporter BCRP. The ensuing downregulation of BCRP compromises placental barrier integrity, rendering trophoblasts vulnerable to the intracellular accumulation of xenobiotics and reactive oxygen species (ROS) (Hit 2: Metabolic Reprogramming) Concurrently, the active HIF-1α/ARNT complex drives a robust glycolytic shift by upregulating a cascade of key transporters and enzymes (GLUT1, HK2, PFK1, LDHA, and PDK1). Notably, elevated PDK1 restricts the entry of pyruvate into the mitochondrial TCA cycle, precipitating mitochondrial dysfunction and excessive lactate accumulation. Collectively, this “dual-hit” cascade—characterized by the loss of barrier defense and pathological metabolic remodeling—orchestrates trophoblast failure, directly contributing to placental insufficiency and the precise pathogenesis of Fetal Growth Restriction (FGR). Abbreviations: AHR, Aryl hydrocarbon receptor; ARNT, Aryl hydrocarbon receptor nuclear translocator; BCRP, Breast cancer resistance protein; FGR, Fetal growth restriction; GLUT1, Glucose transporter 1; HIF-1α, Hypoxia-inducible factor-1α; HK2, Hexokinase 2; LDHA, Lactate dehydrogenase A; PDK1, Pyruvate dehydrogenase kinase 1; PFK1, Phosphofructokinase-1; ROS, Reactive oxygen species; TCA, Tricarboxylic acid.

Integrating our clinical observations (Figure 1) with the *in vitro* molecular data (Figures 2–4), we propose a comprehensive “dual-hit” working model to elucidate how chronic hypoxia orchestrates placental insufficiency (Figure 5).

Under physiological normoxia, the steady-state interaction between AHR and ARNT guarantees the basal transcription of BCRP, thereby maintaining a robust placental barrier against environmental and pharmacological stressors. However, prolonged hypoxia profoundly disrupts this homeostasis. The excessive stabilization and nuclear localization of HIF-1α initiate the first hit (barrier impairment) through competitive protein sequestration. By successfully outcompeting AHR for their shared obligate partner ARNT, HIF-1α dismantles the AHR/ARNT complex. This “disruption of the AHR/ARNT complex” silences AHR signaling, directly collapsing BCRP expression and leaving the trophoblasts vulnerable to xenobiotics and reactive oxygen species (ROS).

Concurrently, the newly formed HIF-1α/ARNT transcriptional machinery dictates the second hit (metabolic reprogramming). It orchestrates a drastic glycolytic shift by systematically upregulating key enzymes (GLUT1, HK2, PFK1, and LDHA). Crucially, the concurrent elevation of PDK1 shunts pyruvate away from the mitochondrial TCA cycle, precipitating severe mitochondrial dysfunction and intracellular lactate accumulation.

Ultimately, this dual-hit cascade—the Substantial loss of pharmacological detoxification coupled with pathological metabolic remodeling—synergistically drives trophoblast failure. This integrated mechanism provides a robust molecular explanation for the impaired placental function and subsequent fetal growth restriction (FGR) clinically observed in early-onset preeclampsia.

## 4 Discussion

### 4.1 Placental insufficiency as the primary clinical driver

The high incidence of fetal growth restriction (FGR, defined as an estimated fetal weight <10th percentile) in our early-onset preeclampsia (PE) cohort underscores the profound and distinct pathology of this phenotype. Unlike late-onset PE, which is predominantly driven by maternal systemic factors, early-onset disease typically originates from defective spiral artery remodeling (Dai et al., 2023; Mason et al., 2011; Dai et al., 2024). This vascular failure engenders a hostile, chronically hypoxic microenvironment that directly dictates placental insufficiency and suppresses fetal growth (Dei et al., 2019). Importantly, while our clinical cohorts exhibited a slight variance in mean gestational age (38.3 vs. 32.8 weeks), our rigorous downstream validations confirm that the distinct molecular signatures observed are fundamentally driven by disease-specific hypoxic stress, rather than physiological placental aging. Collectively, these clinical observations necessitate a comprehensive mechanistic framework—which we propose as a “dual-hit” paradigm—to explain how this hypoxic stress translates into cellular and fetal pathology.

### 4.2 The first hit: substantial metabolic reprogramming and mitochondrial dysfunction

At the cellular level, the trophoblast response to chronic hypoxia is dictated by the aberrant stabilization and persistence of HIF-1α. Under physiological conditions, HIF-1α undergoes degradation as the placenta matures and oxygenates; its pathological persistence in early-onset PE drives a dramatic intracellular remodeling (Wu et al., 2023; Fernández-Torres et al., 2017). Our initial observation of elevated GLUT1 prompted a deeper investigation into this metabolic adaptation. Crucially, our comprehensive functional assays provide definitive evidence of a massive metabolic shift toward hypoxia-driven glycolysis. We demonstrated that hypoxia not only markedly upregulates key glycolytic machinery—including HK2, PFK1, LDHA, and PDK1—but also drives a parallel surge in glucose consumption and lactate accumulation. Concurrently, JC-1 staining revealed a profound Marked reduction in mitochondrial membrane potential, indicating severely compromised oxidative phosphorylation. While this robust glycolytic shift initially serves as an acute compensatory survival mechanism, the resulting intracellular energetic crisis and progressive oxidative stress inevitably disrupt the delicate balance of angiogenic factors (e.g., sFlt-1/PlGF). This primary metabolic “hit” thereby contributes directly to the systemic endothelial dysfunction (John, 2013; Juvale and Wiese, 2015) and impaired villous maturation (Shinde et al., 2025; Larigot et al., 2018) characteristic of early-onset PE.

### 4.3 The second hit: mechanistic crosstalk, ARNT sequestration, and barrier impairment

Parallel to the metabolic crisis, our findings unveil a critical regulatory conflict between cellular survival signaling (HIF-1α) and protective placental barrier function (AHR/BCRP). We found that the crucial efflux transporter BCRP is markedly depleted in preeclamptic placentas, exhibiting a strong inverse correlation with the hypoxic marker GLUT1 (Kojovic et al., 2020; Cygalova et al., 2009). Because AHR strictly requires ARNT as an obligate heterodimeric partner to initiate BCRP transcription (Hu et al., 2023), we elucidated the competitive dynamics between these overlapping pathways.

Our Co-IP assays provided explicit physical evidence of an enhanced HIF-1α/ARNT interaction under hypoxia, while nuclear fractionation demonstrated the subcellular exclusion of AHR. These results mechanistically validate our hypothesis: the hypoxia-induced surge of HIF-1α competitively sequesters available ARNT, effectively dismantling the AHR/ARNT transcriptional complex and silencing BCRP expression.

Crucially, we translated this molecular crosstalk into physiological relevance using the specific BCRP fluorescent substrate, Hoechst 33,342. Exposure to either hypoxia or specific AHR inhibition triggered significant intracellular accumulation of the surrogate toxin, definitively proving that this transcriptional silencing dictates a critical loss of functional efflux capacity. This barrier impairment extends far beyond endogenous oxidative stress; the depletion of BCRP drastically escalates fetal susceptibility to maternal environmental xenobiotics, industrial chemicals, and pharmacological agents. This represents the pivotal second “hit,” highlighting the extreme toxicological vulnerability of the FGR fetus in hypoxic pregnancies (Lye et al., 2019; Anderson et al., 2013; Talari et al., 2018; Jia et al., 2024).

### 4.4 Limitations and clinical implications

Our study has several recognized limitations. First, we acknowledge the significant disparity in mean gestational age (GA) between our PE (32.8 weeks) and control (38.3 weeks) groups, which is a common challenge in studying early-onset PE as it usually necessitates premature delivery [Newly Added Statistical Evidence] To rigorously address this potential confounder, we performed a multivariate linear regression analysis. Crucially, the model identified PE status as a significant independent predictor of BCRP downregulation (β = −0.698,P = 0.042), whereas GA did not show a statistically significant independent effect within this cohort (P = 0.473). While these data strongly support a disease-driven mechanism, we acknowledge that the inclusion of GA-matched spontaneous preterm births in future studies would further refine the distinction between maturational and pathological BCRP regulation.

Second, while the BeWo choriocarcinoma cell line is a robust and widely accepted model that reliably recapitulated the HIF-1α/ARNT competitive axis in our study, immortalized 2D monocultures cannot perfectly replicate the complex, multi-cellular human placental unit *in vivo*. Future investigations incorporating primary human trophoblast organoids or trophoblast-specific *in vivo* knockout models are warranted to further substantiate these findings.

Nonetheless, our integrative approach—bridging clinical cohort data, intracellular physical interactomics, and real-time functional efflux assays—establishes a rigorous “dual-hit” pathological framework. Clinically, unraveling the vulnerabilities within this HIF-1α/AHR/BCRP competitive axis provides crucial insights into placental insufficiency syndromes. It underscores the urgent need for stringent xenobiotic management in pregnancies complicated by early-onset PE, and suggests that therapeutically protecting AHR signaling or modulating placental hypoxia could represent a novel Frontier to safeguard fetal development in FGR.

## Data Availability

The datasets generated and/or analyzed during the current study are not publicly available due to protecting participant privacy, but are available from the corresponding author on reasonable request.
