# Correction: Hypoxia-induced metabolic shifting and AHR/BCRP axis impairment drive placental dysfunction in early-onset preeclampsia and FGR

**DOI:** 10.3389/ftox.2026.1953267

**Published:** 2026-09-09

**Authors:** Jingjing Shi, Wenxuan Zhu, Haixia Liu, Ping Yang, Yongzhong Gu, Wen Peng, Jinlai Meng

**Affiliations:** 1 Department of Obstetrics and Gynecology, Shandong Provincial Hospital Affiliated to Shandong First Medical University, Jinan, Shandong, China; 2 Jining Medical University, Jining, Shandong, China; 3 Key Laboratory of Maternal & Fetal Medicine of National Health Commission of China, Shandong Provincial Maternal and Child Healthcare Hospital Affiliated to Qingdao University, Jinan, Shandong, China; 4 Department of Obstetrics and Gynecology, Shandong Provincial Hospital, Shandong University, Jinan, Shandong, China

**Keywords:** aryl hydrocarbon receptor (AhR), breast cancer resistance protein (BCRP), fetal growth restriction (FGR), hypoxia-inducible factor 1 alpha (HIF-1α), placental barrier, preeclampsia

The funder “Special Fund of Shandong Provincial Medical Association”, YXH 2022DZX06009 to Jinlai Meng was erroneously omitted.

The original version of this article has been updated.

